# Cerebellar transcranial magnetic stimulation improves motor function in Parkinson's disease

**DOI:** 10.1002/acn3.52183

**Published:** 2024-09-05

**Authors:** Marcus Grobe‐Einsler, Viktoria Baljasnikowa, Aline Faikus, Tamara Schaprian, Oliver Kaut

**Affiliations:** ^1^ Department of Neurology University Hospital Bonn Bonn Germany; ^2^ German Center for Neurodegenerative Diseases (DZNE) Bonn Germany; ^3^ SRH Gesundheitszentrum Bad Wimpfen GmbH Bad Wimpfen Germany

## Abstract

**Objective:**

To determine whether an accelerated protocol of 48 Hz cerebellar repetitive transcranial magnetic stimulation results in improved motor function in individuals with Parkinson's disease.

**Methods:**

In this double‐blind randomized sham‐controlled study, 35 individuals with Parkinson's disease and stable medical treatment were randomized to either sham or verum transcranial magnetic stimulation. The stimulation was applied bilaterally and medial over the cerebellum and comprised a novel accelerated protocol encompassing two sessions per day on 5 consecutive days. Patients were assessed at baseline, on day 5 after the last stimulation and 1 month post intervention. Measurements included dynamic posturography, UPDRS III, 8‐Meter walk test, and Timed Up and Go test.

**Results:**

The accelerated protocol was safe and feasible in an outpatient setting. Patients in the verum group showed significant improvement (*p* < 0.001) of motor symptoms as measured in the UPDRS III. Improvement was mainly carried by the domains rigor, bradykinesia, and gait and persisted after 1 month (*p* = 0.009), whereas tremor remained unchanged.

**Interpretation:**

The effect of a high‐dose transcranial magnetic stimulation in patients with Parkinson's disease is encouraging and comparable to other studies using much longer stimulation protocols. This short‐term intervention of 5 days facilitates the future application in an outpatient setting. Reduction in hospitalization rates directly benefits patients with motor impairment.

## Introduction

Pharmacological treatment of Parkinson's disease (PD) is effective, but limited by side effects. Nonpharmacologic treatment includes physiotherapy, and physical and speech therapy with limited success rates.^1^ Thus, there is a need for complementary therapeutic options with less adverse events. Repetitive transcranial magnetic stimulation (rTMS) is a promising technique for noninvasive brain stimulation with positive effects on many neurological disorders including PD. It is well tolerated, painless and has only minimal side effects.^2^ The duration of classic stimulation protocols and correspondingly delayed treatment effects limit feasibility. To address this problem and based on the assumptions that repeated TMS application with a greater total number of pulses leads to equal or even greater treatment effects and that condensed treatment duration has a durable effect, accelerated protocols were developed. To date there are no published protocols for accelerated TMS in PD patients. The most extensively researched TMS protocols can be found for depression, as there is a marketing authorization and guideline recommendation for this indication. Despite heterogeneity in the applied stimulation protocols with 15,000 to 90,000 stimuli applied during 2–10 daily sessions with intersession intervals ranging from 12 min to 12 h, meta‐analyses suggest that accelerated protocols improve depressive symptoms more rapidly.^3^, ^4^ The accelerated protocols were also demonstrated to be safe, effective, and well tolerated in patients with major depressive episode.^5^, ^6^, ^7^, ^8^ In the field of movement disorders, the primary motor cortex (M1) is the most common target location for TMS in PD patients.^9^ While PD is primarily characterized by neurodegeneration in the nigrostriatal system resulting in a dopaminergic deficit, growing evidence now suggests pathophysiological involvement of the cerebellum.^10^, ^11^, ^12^ For example, a number of studies demonstrated increased cerebellar activity and connectivity in PD patients^11^, ^13^, ^14^ whereas cerebellar connectivity with the basal ganglia seems to be reduced in later disease stages.^15^, ^16^, ^17^ This relationship may be indicative of a mechanism to bypass the impaired basal ganglia function that collapses with progression of disease.^13^, ^18^, ^19^, ^20^, ^21^ The exact pathophysiological role of the cerebellum in PD is yet to be explored, but the potential involvement makes the cerebellum an interesting therapeutic target for TMS in PD patients. To date, studies on cerebellar TMS in PD are seldom. Koch and colleagues report amelioration of levodopa‐induced dyskinesia after a single unilateral continuous theta burst stimulation (TBS) session of the cerebellar hemisphere in 10 PD patients. An extended stimulation protocol of 2 weeks daily stimulation in 20 patients led to prolonged treatment effects for up to 4 weeks.^22^ These findings were later confirmed by another study.^23^ Bologna and colleagues later demonstrated reduced excitability over contralateral M1 after unilateral continuous TBS of the cerebellar hemisphere, but found no beneficial effects on resting tremor in 13 PD patients.^24^ Furthermore, freezing of gait was not improved after continuous or intermittent cerebellar TBS in 17 PD patients in a cross‐over design.^25^

If the increased cerebellar connectivity in PD patients is a compensation for impaired basal ganglia function and resultant motor deficits, we hypothesize that stimulation of the cerebellum using rTMS may enhance the putative cerebellar compensation resulting in improved motor function in PD patients. Consequently, we have chosen the cerebellum as stimulation target in our study. To optimize feasibility of rTMS, we developed an accelerated protocol with two daily sessions on 5 consecutive days. The aim of this study was to demonstrate feasibility and investigate treatment effects of accelerated cerebellar rTMS in PD patients in an outpatient setting.

## Methods

### Patients

Thirty‐five patients with the diagnosis of idiopathic PD according to the UK Brain Bank Criteria^26^ were enrolled consecutively from the Department of Neurology, University Clinic Bonn, Bonn, Germany. The inclusion criteria were age of 40 years or older and stable PD therapy for at least 1 month. Exclusion criteria were a history of seizures, frequent headaches, head injury or any neurosurgical intervention, dementia, treatment with neuroleptics, the presence of metallic particles in the head, cardiac pacemakers or neurostimulators, and changes in PD medication during the trial.

As part of the patient characterization, patients were interviewed about history of falls during the past year and classified as faller or nonfaller.^27^ A fall was defined as “unintentionally coming to rest on the ground, floor, or other lower level”.^28^ Subjects were classified as fallers if they reported that they had fallen more than once during the previous 12 months.^29^

**Figure 1:**
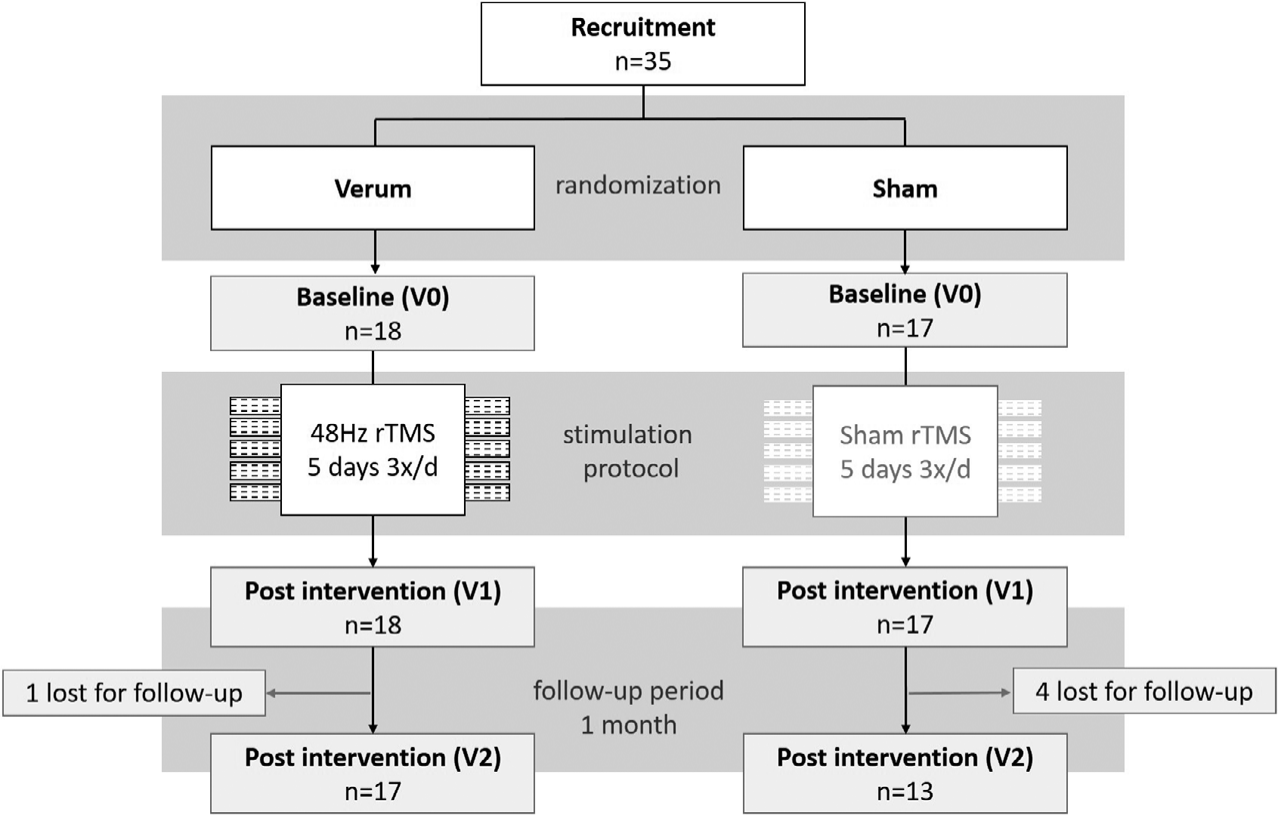
Study protocol.

This study was performed in accordance with the Code of Ethics of the World Medical Association (Declaration of Helsinki) and approved by the Ethics committee of the University hospital of Bonn, Germany (No. 209/20, date 06/02/2020). All patients gave written informed consent for participation. This trial was registered at the German Clinical Trial Registry https://drks.de (DRKS‐ID: DRKS00022356); the flowchart of the study design is shown in Figure 1.

### Study design

Studies on cerebellar TMS in PD are seldom, previous studies report sample sizes between 10 and 20 patients, and the authors report no effect sizes that could be used for robust sample size calculations.^22^, ^23^, ^24^, ^25^ Based on these studies, we assumed that a sample size of 15 patients per group would suffice to reach an acceptable power. The study design is double‐blind, randomized and sham‐controlled, with a 1:1 allocation ratio. Participants were allocated to either the verum (rTMS 48 Hz) or the control group (sham) using a block randomization with an AAABBB distribution model (A = experimental; B = control). Treatment allocations were kept in sequentially numbered envelopes and opened only at the time of enrolment. The medication remained unchanged throughout the study, and stimulation was administered during the “on medication” state.

### Clinical assessments

Patients were assessed at baseline (V0), at the end of the treatment session (V1 at day 5) and at the end of the trial (V2 at day 30) during the on medication state. Assessments included a dynamic posturography, the Unified PD Rating Scale, part III (UPDRS III), the 3 meter “Timed Up and Go” (TUG) test, and the timed 8‐meter walk test (8MW). Improvement in dynamic posturography between V0 and V1 was selected as primary outcome measure. Secondary outcome measures were reduction in UPDRS III, times for TUG and 8MW, and reduction in the UPDRS III subdomains face and speech (Items 1–2), rigor (Item 3), bradykinesia (Items 4–8 and 14), gait (Items 9–11), postural stability (Item 12), posture (Item 13), and tremor (Items 15–18).

### Intervention

To strengthen feasibility outside a study setting, the rTMS intervention consisted of 10 sessions (two sessions per day) over 5 consecutive days (e.g., Monday to Friday). We chose a figure‐of‐eight coil, because this coil shape was previously chosen for studies investigating effects of cerebellar TMS^22^, ^24^, ^25^ and the coil shape has been shown to effectively modulate the cortical excitability of the contralateral motor cortex after cerebellar stimulation.^22^, ^30^

A Magstim Rapid2 (Magstim Co. Ltd, Whitland, UK) with an air‐cooled figure‐of‐eight coil delivered stimuli with the coil held tangentially over three regions, always in the same order: Starting with the left lateral cerebellum (with the coil located 4 cm left to the inion), followed by the median cerebellum (coil located on inion) and then the right lateral cerebellum (coil located 4 cm right to the inion). The choice of cerebellar hemispheres as stimulation targets was influenced by previous cerebellar TMS studies in PD patients.^22^, ^23^, ^24^, ^25^ We additionally included the vermis due to its pathophysiological involvement in postural control. Sham treatment was performed with a Magstim AFC sham coil that looks identical to its active version, replicates operational sounds, and delivers a very shallow magnetic field to mimic the sensation of magnetic stimulation. The sham coil was installed by an unblinded investigator and neither patients, nor blinded investigators were able to identify which coil is sham or verum. During stimulation, patients were provided with earplugs and laid their head down on a pillow on a small table in front of them, and the TMS coil was held facing upward. The coil was held in place, touching the patient's head, with a mechanical arm. An investigator was present during the stimulation to correct the position of the mechanical arm if necessary and ensure continuous contact of the TMS coil to the head. For setup and stimulation targets, see Figure S1.

Technical specifications of the TMS device cause reduction in stimulation power for frequencies >50 Hz. The experimental group therefore received rTMS similar to a standard intermittent TBS paradigm consisting of three stimuli bursts at 48 Hz, in contrast to standard protocols consisting of 50 Hz, repeated at 5 Hz frequency. Previous studies on cerebellar TMS in PD patients report applies stimulation intensities between 70 and 80% of MT over M1.^22^, ^23^, ^24^, ^25^ However, due to a lack of evidence that individual adjustment of TMS power via determination of motor threshold over M1 correlates with clinical outcome after stimulation in another brain region, we believe that assessment of the motor threshold is dispensable in this case. Therefore, all patients were stimulated with a fixed output intensity of 50% of maximum stimulator output intensity. One train consisted of 15 bursts with a 5.4 sec inter‐train interval, for each region 32 trains were delivered. Stimulation sessions were done hourly, two sessions per day: One session lasted approximately 15 min (3 min for stimulation on each site and approximately 3 min for repositioning of the coil for the next stimulation site). Intersession intervals were 45 min. All stimulations were performed in the afternoon, the stimulation on the following days took place at the same time ± 2 h.

### Statistical analysis

All analyses were performed using R Software for Statistical Computing, version 4.2.1 R Foundation for Statistical Computing, Vienna. Two‐way mixed ANOVAs were performed to evaluate the effects of treatment (verum and sham) and visits (V0, V1, V2) on UPDRS III, UPDRS subcategories, TUG, 8MW, and posturography. The assumptions for a two‐way mixed ANOVA were checked and were not violated. Post hoc tests for pairwise comparisons were performed using pairwise *t*‐tests. Descriptive statistics were performed for scores and demographic data.

## Results

Patient characteristics were similar in both groups with no significant differences between the outcome measures at baseline. Detailed descriptive statistics are shown in Table 1. For comparison of baseline characteristics between groups, see Table S1. Patients in the verum group were slightly younger (66 years (SD = 10) in the verum group versus 70 years (SD = 10) in the sham group), but mean disease duration was 7 years in both groups (SD = 6 in the verum group, and SD = 5 in the sham group). The patients in the verum group had slightly higher disease severity as measured in UPDRS III (34 points in the verum versus 30 points in the sham group), but the mean Hoehn & Yahr stage was 2 in both groups and patients in the verum group had a lower daily levodopa dose (331 mg/d versus 388 mg/d). In total, 17% of the patients (*n* = 3) in the verum group and 12% of the patients (*n* = 2) in the sham group did not take any levodopa. In total, 44% of the patients in the verum group (*n* = 8) were categorized as fallers versus 29% (*n* = 5) in the sham group. Women were underrepresented in both groups (31% (*n* = 3) female in the verum group, 20% (*n* = 4) female in the sham group).

**Table 1:** Patient characterization and progression of outcome parameters, reported as mean (SD), after intervention (verum or sham).

	Sham	Verum
Baseline characteristics		
*n*	17	18
Female	4	3
H&Y stage	1.94 (0.75)	2.11 (0.90)
Daily levodopa [mg]	388 (248)	331 (195)
Age	70.41 (10.37)	66.06 (9.70)
AOO	63.35 (12.15)	59.22 (10.42)
Falls (12 months)	0.31 (0.48)	0.39 (0.50)

Visit	V0	V1	V2	V0	V1	V2
Posturo	251.87 (72.52)	221.31 (60.51)	231.58 (42.59)	254.78 (58.27)	242.61 (61.29)	257.00 (60.19)
UPDRS	30.12 (14.65)	31.81 (11.72)	32.92 (12.59)	34.17 (14.02)	29.39 (13.14)	30.53 (12.87)
UPDRS subdomains						
Face and speech	2.75 (1.06)	2.75 (1.06)	2.75 (1.14)	2.61 (1.04)	2.61 (1.04)	2.71 (0.99)
Rigor	5.25 (2.74)	5.94 (2.67)	6.17 (2.62)	5.94 (3.15)	4.89 (2.61)	5.65 (2.62)
Bradykinesia	12.44 (5.85)	12.25 (4.75)	13.33 (5.63)	15.61 (7.18)	13.11 (6.43)	13.00 (6.43)
Gait	2.12 (1.63)	2.06 (1.48)	1.92 (1.44)	2.44 (1.89)	2.06 (1.55)	2.00 (1.58)
Postural stab	1.62 (1.15)	1.44 (1.21)	1.75 (1.29)	1.22 (1.22)	1.28 (1.27)	1.35 (1.22)
Posture	1.44 (0.63)	1.44 (0.63)	1.33 (0.49)	1.50 (1.10)	1.50 (1.10)	1.47 (1.18)
Tremor	6.38 (5.48)	5.94 (5.12)	5.67 (5.65)	4.83 (4.71)	3.94 (4.18)	4.35 (4.62)
TUG	8.98 (2.89)	8.05 (2.29)	9.29 (2.92)	9.95 (5.28)	9.11 (5.46)	9.22 (5.98)
8MW	5.71 (1.60)	5.80 (1.60)	6.28 (2.24)	5.75 (1.93)	5.37 (1.54)	5.64 (2.19)

8MW, 8‐meter walk test; AOO, age of onset; Postural stab, postural stability; Posturo, dynamic posturography; TUG, Timed Up and Go test.

All randomized patients completed the stimulation protocol in their group and V0 and V1 assessments. Only minor adverse events were reported during or after stimulation. Interestingly, headache was more common in the sham group. Overall, slight headache after stimulation, fatigue after stimulation, and muscle tension in the head and neck region after stimulation were each reported by 11% (*n* = 2) of the patients in the verum group. In the sham group, these symptoms were reported by 23% (*n* = 4), 18% (*n* = 2), and 6% (*n* = 1), respectively. Discomfort or pain during stimulation was reported by 22% (*n* = 4) of the patients in the verum group and by none of the patients in the sham group. None of the minor adverse events led to deviation in the study protocol. 95% of the patients in the verum group (*n* = 17), and 76% of the patients in the sham group (*n* = 13) completed V2 assessments after end of rTMS.

**Figure 2:**
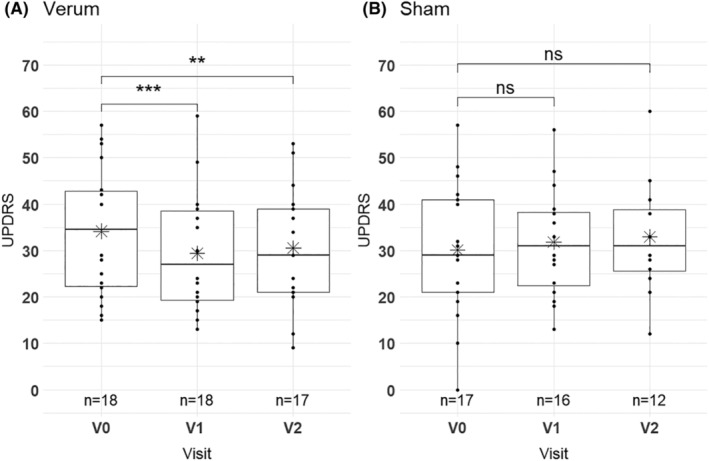
Boxplots of UPDRS scores for verum (A) and sham (B) group. Scores were obtained before rTMS stimulation (V0), after last therapy session (V1) and 1 month later (V2). The star symbols present the mean values of sham and verum visits, results of group comparisons are shown as ns (not significant), ** for *p* ≤ 0.01 and *** for *p* ≤ 0.001.

Results from ANOVA analysis for all outcome measures are shown in Table 2. For the dynamic posturography, ANOVA showed no significant interaction effect between treatment and visits (*F* (2, 54) = 0.941, *p* = 0.396) and no significant simple main effect of treatment (*F* (1, 27) = 1.105, *p* = 0.302). There was a significant simple main effect of visits (*F* (2, 54) = 5.391, *p* = 0.008) (Table 2) and pairwise comparison showed only a significant effect of V0 vs. V1 (*p*.adj = 0.001) (Table 3). rTMS significantly improved disease severity as measured by UPDRS III between V0 and V1 in the verum group only: The results of the ANOVA indicated a significant interaction effect between treatment and visits (*F* (2, 54) = 9.767, *p* ≤ 0.001, see Figure S2) and a significant main effect of visits (*F* (2, 54) = 4.036, *p* ≤ 0.001). There was no significant simple main effect of treatment (*F* (1, 27) = 0.0003, *p* = 0.960). Considering the Bonferroni adjusted *p*‐value (*p*.adj) of pairwise comparison, the simple main effect of visits on UPDRS III was significant only for the verum group (*p* ≤ 0.001), but not for the sham group (*p* = 0.260) (Fig. 2 and Table 4). The post hoc pairwise comparisons of UPDRS III showed a significant reduction between V0 and V1 (*p*.adj ≤0.001) that persisted until V2 (V0 vs V2 *p*.adj = 0.009). Hence, UPDRS III was not significantly different between V1 and V2 in the verum group (Table 5). The mean UPDRS III values were significantly higher on V0 (mean = 34.17, SD = 14.02) than on V1 (mean = 29.39, SD = 13.14) or V2 (mean = 30.53, SD = 12.87) for verum group (Tables 1 and 5). In the sham group, the post hoc pairwise comparison showed no significant reduction in UPDRS III (Table 5). There were no significant main and interaction effects for the secondary outcome measures 8MW (*F* (2, 54) = 0.363, *p* = 0.394) and TUG (*F* (2, 54) = 1.208, *p* = 0.307) in both groups (Table 2).

**Table 2:** Results of two‐way mixed ANOVA: Simple main effects of treatment and visits, and interaction effects (treatment:visit).

	Effect	DFn	DFd	*F*‐value	*p*‐value	ges
Posturo	Treatment	1	27	1.105	0.302	0.035
	Visit	2	54	5.319	**0.008**	0.021
	Treatment:visit	2	54	0.941	0.396	0.004
UPDRS	Treatment	1	27	0.003	0.958	0.000102
	Visit	2	54	4.360	**0.018**	0.006
	Treatment:visit	2	54	9.770	**<0.001**	0.013
UPDRS subscores						
Face and speech	Treatment	1	27	0.012	0.912	0.001
	Visit	2	54	na	na	na
	Treatment:visit	2	54	na	na	na
Rigor	Treatment	1	27	0.013	0.908	0.001
	Visit	2	54	1.586	0.214	0.006
	Treatment:visit	2	54	4.036	**0.023**	0.015
Bradykinesia	Treatment	1	27	0.471	0.499	0.016
	Visit	2	54	1.980	0.148	0.007
	Treatment:visit	2	54	6.089	**0.004**	0.021
Gait	Treatment	1	27	0.256	0.617	0.009
	Visit	2	54	3.982	**0.024**	0.005
	Treatment:visit	2	54	3.982	**0.024**	0.005
Postural stab	Treatment	1	27	0.386	0.540	0.013
	Visit	2	54	0.754	0.476	0.002
	Treatment:visit	2	54	0.624	0.540	0.002
Posture	Treatment	1	27	0.253	0.619	0.009
	Visit	2	54	0.689	0.502	0.000
	Treatment:visit	2	54	0.689	0.502	0.000
Tremor	Treatment	1	27	0.493	0.489	0.017
	Visit	2	54	2.146	0.127	0.002
	Treatment:visit	2	54	0.716	0.493	0.001
MW8	Treatment	1	27	0.363	0.552	0.012
	Visit	2	54	0.826	0.443	0.004
	Treatment:visit	2	54	0.949	0.394	0.004
TUG	Treatment	1	27	0.177	0.678	0.006
	Visit	2	54	2.659	0.079	0.008
	Treatment:visit	2	54	1.208	0.307	0.004

*F* values are not available (na), when variability of parameters is too low to calculate ANOVA. Bold *p*‐values are significant.

8MW, 8‐meter walk test; DFd, degree of freedom for the denominator; DFn, degree of freedom for the numerator; ges, generalized Eta^2^ measure of effect size; Postural stab, Postural stability; TUG, Timed Up and Go test.

**Table 3:** Results of post hoc pairwise comparisons of dynamic posturography (*t*‐statistic).

Visits	*p*	*p*.adj
V0, V1	**0.0004**	**0.001**
V0, V2	0.601	0.999
V1, V2	0.036	0.109

Bold *p*‐values are significant. *p*‐value (*p*), Bonferroni corrected *p*‐values (*p*.adj). Baseline visit (V0) and first follow‐up visit directly after last sham or verum rTMS stimulation (V1) and after 1 month (V2).

**Table 4:** Results of pairwise comparisons for the main effect of visit for UPDRS.

Treatment Effect	DFn	DFd	*F*	*p*	ges	*p*.adj
Sham (visit)	2	22	2.24	0.13	0.004	0.26
Verum (visit)	2	32	11.8	**<0.001**	0.031	**<0.001**

Bold *p*‐values are significant. DFn (degree of freedom for the numerator), DFd (degree of freedom for the denominator), ges (generalized Eta^2^ measure of effect size), and *p*.adj (Bonferroni corrected *p*‐values).

**Table 5:** Results of post hoc pairwise comparisons of UPDRS (*t*‐statistic).

Treatment	Visits	*p*	*p*.adj
Sham	V0, V1	0.817	1
	V0, V2	0.13	0.39
	V1, V2	0.095	0.285
Verum	V0, V1	**<0.001**	**<0.001**
	V0, V2	**0.003**	**0.009**
	V1, V2	0.863	1

Bold *p*‐values are significant. *p*‐value (*p*), Bonferroni corrected *p*‐values (*p*.adj). Baseline visit (V0) and first follow‐up visit directly after last sham or verum rTMS stimulation (V1) and after 1 month (V2).

To identify which domains of UPDRS III contributed most to overall reduction in the score, UPDRS III was divided into the subscores face and speech (Items 1–2), rigor (Item 3), bradykinesia (Items 4–8 and 14), gait (Items 9–11), postural stability (Item 12), posture (Item 13), and tremor (Items 15–18). The results of the ANOVA for UPDRS III subscores showed a significant interaction effect between treatment and visits for rigor (*F* (2, 54) = 4.036, *p* = 0.023), bradykinesia (*F* (2, 54) = 6.089, *p* = 0.004), and gait (*F* (2, 54) = 3.982, *p* = 0.024) in the verum group, but not for the subscores postural stability (*F* (2, 54) = 0.624, *p* = 0.540), posture (*F* (2, 54) = 0.689, *p* = 0.502), and tremor (*F* (2, 54) = 0.716, *p* = 0.493). Variability in the face and speech subscore was too low to calculate ANOVA (Table 2).

The small sample size in the subgroups of fallers and nonfallers did not allow for robust statistical testings. In an exploratory analysis, both subgroups showed improvement of PD symptoms between V0 and V1 in the verum group with a trend toward greater effect in the subgroup of fallers. Reduction in UPDRS III was 11.5% (−3.1 points) in the subgroup of nonfallers and 15.9% (−6.87 points) in the subgroup of fallers, TUG reduced by 6.3% (−0.44 s) in the nonfallers compared to 9.8% (−1.35 s) in the fallers subgroup, and 8MW improved by 3.2% (−0.15 s) in the nonfallers compared to 9.6% (−0.66 s) in the fallers subgroup.

A power analysis based on the two‐way mixed ANOVA with repeated measures of UPDRS III resulted in a statistical power of 0.55 at a significance level of *α* = 0.1 and an effect size of f = 0.12 (see Fig. S3). Based on this power analysis, future studies would need >25 patients per group to achieve a power of 70%.

## Discussion

In this study, we demonstrated that repetitive transcranial magnetic stimulation can improve motor function in PD as measured in UPDRS III in a randomized controlled trial design, although we did not observe an improvement in the primary outcome measure dynamic posturography. In the treatment group, the UPDRS III sum score improved significantly by 4.78 points (14.0%) after 5 days of cerebellar stimulation whereas the sham group showed no improvement. This effect persisted for at least 4 weeks after the intervention, but larger sample sizes are desirable for future studies to achieve a higher statistical power.

### Effect of rTMS on PD motor symptoms

Standard medical therapy alone yields a mean UPDRS III reduction of 27.4%.^31^ Our study population comprised PD patients with optimized and stable medical therapy. In this population, UPDRS III improved by another 14.0% after cerebellar rTMS. The therapeutic effect of rTMS is smaller than best medical treatment alone, but with a favorable profile of side effects. Our study lacks a control group without medical therapy, but we assume that best medical therapy and TMS are complementary and the combination of both yields best therapeutic effects. Additional intensified physiotherapy is likely to further improve therapeutic effects: Chung and colleagues report a reduction in UPDRS III by 8 points after 25 Hz stimulation of M1 in PD patients in combination with treadmill training.^32^

In our study, reduction in UPDRS III was mainly carried by improvements in the domains rigor, bradykinesia and gait, whereas posture, postural stability and tremor remained unchanged. These findings are in line with a study by Bologna and colleagues, who found no reduction in tremor after cerebellar TMS assessed by objective kinematic techniques.^24^

Results of TMS on postural stability are inconclusive. In patients with spinocerebellar ataxia (SCA), cerebellar rTMS showed positive effects on postural control and gait.^33^ In our study, this effect could not be transferred to PD patients and postural stability remained unchanged. On the other hand, a recent meta‐analysis found no improvement of postural stability after rTMS activation of M1 or the supplementary motor area, which is in line with our findings.^34^

Unexpectedly, the significant improvement of gait function as measured by UPDRS III did not lead to significant improvements in the secondary outcome measures 8MW and TUG. While these assessments could be considered more objective due to the measurement of speed as single outcome, the UPDRS III gait subdomain incorporates more complex parameters other than speed (UPDRS III Items 3.9–3.11) and thus cannot be directly compared with TUG and 8MW. Changes in the gait subdomain may therefore still be of functional relevance, even if gait speed remains unchanged.

Falls are frequently reported by PD patients and represent a major determinant of reduced quality of life.^35^ This study was not designed for subgroup analysis, and sample sizes were too small for sophisticated statistical methods in that regard. However, an exploratory comparison of outcome parameters in PD patients with and without frequent falls (“fallers” versus “nonfallers”) showed a tendency toward higher treatment effects in the subgroup of fallers. The highest improvement rates were observed in the 8MW after treatment (9.5% reduction in the fallers versus 3.2% in the nonfallers subgroup), but the absolute change was only −0.66 s for the 8MW. Functional meaningfulness of such small changes is questionable, but based on this exploratory analysis, we assume that treatment effects may be greater in more severely affected patients and future studies should focus on this subgroup.

To date PD studies, investigating cerebellar stimulation protocols are rare. Janssen and colleagues observed changes in gait speed after theta burst stimulation (TBS) of one cerebellar hemisphere in PD patients with freezing of gait.^25^ Other studies demonstrated positive effects of cerebellar TBS on levodopa‐induced dyskinesia, but without significant improvement of UPDRS scores.^22^, ^23^

### Impact of stimulation protocol

We assume that the positive effects on bradykinesia, rigidity, and gait in this study are at least in part derived from the novel intensified stimulation protocol. The medial cerebellum, including the vermis, is known to be involved in gait and balance control.^36^, ^37^, ^38^ Consequently, the medial cerebellum has been included in TMS stimulation protocols in ataxia patients with positive effects on stance and gait.^39^, ^40^, ^41^ Although the medial cerebellum has been proposed as target location for TMS stimulation protocols in PD,^25^ inclusion of this area in stimulation protocols in PD patients is still rare.^42^, ^43^ To our knowledge, this is the first study with stimulation of both cerebellar hemispheres and the medial cerebellum in PD patients. However, our study design does not allow for determination of optimal TMS target locations, since all patients were stimulated at the same locations (both cerebellar hemispheres and medial cerebellum). Our hypothesis that the positive effects described in this study are (in part) attributable to stimulation of the medial cerebellum therefore cannot be confirmed at this stage.

When applying TMS, the induced electric field strength decreases with increasing distance from the coil.^44^ The anatomical scalp‐to‐cortex distance therefore has considerable influence on the reach of the applied coil and TMS is limited to superficial cortical targets, around 2–3 cm in depth.^45^ Due to the deeper location of the vermis (scalp‐to‐cortex distance of approximately 3 cm with considerable variability), we cannot rule out that the positive effects observed in our study are solely based on stimulation of the cerebellar hemispheres.

Therapeutic effects of rTMS increase with greater numbers of pulses.^46^, ^47^ In addition, multiple stimulation sessions per day with intersession intervals of 50–90 minutes have a cumulative effect on synaptic strengthening.^48^, ^49^ TMS is a standard therapy in patients with depression and safety and effectiveness was demonstrated for stimulation protocols with up to 10 sessions per day in these patients.^5^ Our study utilizes a novel stimulation protocol suitable for patients with movement disorders with a tremendous expansion of the total number of rTMS pulses condensed in 10 sessions over 5 days.

Due to a lack of evidence that individual adjustment of TMS stimulation power correlates with clinical outcome, we believe that determination of the motor threshold is dispensable. For this reason, all patients in this study were stimulated with the same output intensity. This modification further improves feasibility in the outpatient clinical setting. Our study demonstrates feasibility, tolerability, safety, and effectiveness of the novel stimulation protocol in PD patients in an outpatient setting. The profile of side effects and tolerability were good with only few reported minor adverse events.

The significant improvement of PD symptoms after only 5 days of rTMS is remarkable, because most other stimulation protocols include a treatment duration of at least 2 weeks. We hypothesize that further extension of the protocol duration with a consecutively higher number of stimulations could booster the improvement of motor function.

### Putative neurophysiological mechanisms underlying the observed changes

The number of neurons in the cerebellum outnumbers those of the cerebral cortex and basal ganglia and the cerebellum has complex connections to numerous other areas of the brain.^50^, ^51^ While previously, cerebellar function has mainly been linked to motor coordination, execution, and motor learning, it is now also recognized to play a role in cognition, emotion, and behavior.^52^, ^53^, ^54^, ^55^ The role of the cerebellum in PD is still incompletely understood, but we are constantly gaining new insights into the complex interactions through which the cerebellum may influence PD symptoms. For example, the discovery of disynaptic connections between the cerebellum and the basal ganglia suggests that basal ganglia and cerebellar circuits can act independently from the level of the cerebral cortex.^52^, ^56^

Formaggio and colleagues demonstrated that TMS of the primary motor cortex normalized dysfunctional brain oscillations in PD patients.^57^ A reset of the same dysfunctional beta oscillations in PD patients could be induced by cerebellar TMS, for example via the cerebello‐thalamo‐cortical pathway in PD. Whether this mechanism is involved in the observed changes in this study remains to be explored.

Our initial hypothesis that increased cerebellar activity in early stage PD is indicative of compensatory role of the cerebellum that collapsed in later disease stages^13^, ^18^, ^19^, ^20^, ^21^ is supported by experiments on the role of the cerebellum in motor learning: Koch and colleagues found that cerebellar iTBS increased cortical activation during visuo‐adaptive motor tasks and improved error reduction in healthy subjects.^58^ We assume that improved motor learning, induced by cerebellar TMS, may have compensated for acquired motor deficits in PD in our study, but the exact mechanisms underlying the observed changes remain to be explored.

### Limitations

This study was designed to investigate treatment effects of the proposed rTMS stimulation protocol. As reports from previous studies on cerebellar TMS in PD do not allow for robust sample size calculations, we conducted a post hoc power analysis based on the results of this study. The achieved power is sufficient to detect effects of a magnitude of α = 0.1 with a probability of 55%. While this may be acceptable to detect the effects under investigation, higher statistical power is desirable for future studies to optimize reliability of the results and detect smaller changes. Utilizing results from underpowered studies involves critical reflection, identifying trends, and generating further research questions. Despite statistical limitations, qualitative insights offer a starting point for future investigations. According to our post hoc power analysis, future studies should aim to recruit at least 25 patients per group to achieve a power of 70%. Adjustments of recruitment criteria and analysis strategy could potentially further enhance the robustness of study findings. The exploratory post hoc subgroup analysis is indicative of larger therapeutic effects in the subgroup of fallers, which may help to determine optimal recruitment criteria for future studies. However, as discussed, the subgroup analysis is purely exploratory, as are conclusions on possible selection criteria for future TMS studies in PD patients.

This study was designed double‐blind. The sham coil looks identical to its active version, replicates operational sounds, and mimics magnetic stimulation by shallow magnetic fields. However, blinding success was not assessed, which leaves a chance of possible placebo or expectation effects.

Despite the good rationale for involvement of the vermis in gait function in PD patients, the medial cerebellum is not routinely included in TMS stimulation in PD patients. In our study, all patients received stimulation in both cerebellar hemispheres and the medial cerebellum. Due to the deep location of the vermis, we cannot confirm that the TMS field reached the vermis in every patient. This study therefore does not allow for identification of the most effective target location, and we cannot rule out that a unilateral stimulation or sole stimulation of the cerebellar hemispheres would have been sufficient. Furthermore, stimulation of the cerebellum was performed using landmarks. Application of neuro‐navigation would likely have optimized consistency of target location within and across sessions and the ability to target based on individual brain anatomy. This is a limitation of the study protocol.

Another limitation derives from the fixed stimulation intensity that was used for all patients. Even if electric field estimation indicates that the induced electric field from our study protocol can principally reach the target location (Fig. S4), we cannot rule out that anatomical variability led to insufficient stimulation in some individuals, especially in the median cerebellum, due to its deep location. As described in the methods, we believe that determination of the motor threshold over M1 for selection of stimulation power in a distinct brain region is arbitrary, but this problem could be resolved by determination of the necessary stimulation intensity via measurement of cerebellar inhibition.^59^ However, this approach is more time‐intensive. Furthermore although figure‐of‐eight coils are frequently used for cerebellar TMS stimulation, other coil shapes may be more effective in inducing therapeutic effects in future studies.

All observed treatment effects persisted until V2 (1 month after last stimulation day of TMS). The true duration of treatment effect therefore remains to be determined in future studies with longer follow‐up periods.

The traditional view of cerebellar functions as being involved in only primarily motor‐related processes has changed. There is a broad agreement about the involvement of the cerebellum in diverse domains of emotional processing like emotional perception and recognition, evaluation of emotional context and integration into social behavior.^60^ Furthermore, evidence from neuroimaging and patient populations suggests that the cerebellum supports cognitive function. Accordingly, individuals with focal cerebellar lesions show a complex pattern of cognitive and affective deficits termed “cerebellar cognitive affective syndrome”.^55^, ^61^ As a limitation of our study, we did not assess the cognitive and emotional state at baseline and above all, after treatment. Thus, we cannot conclude on possible effects of cerebellar rTMS on cognitive or emotional functions.

## Conclusions and Clinical Implications

To conclude, we demonstrate that cerebellar rTMS using an accelerated 5‐days stimulation protocol improves motor function in PD patients, but did not improve postural stability as measured by dynamic posturography. Future studies should aim for larger sample sizes of >25 patients per group to increase statistical power. The treatment effect is mainly carried by the UPDRS III subdomains rigor, bradykinesia, and gait. We extended previous stimulation protocols showing that an ultra‐short and high‐intensive intervention is tolerable, feasible with a good safety profile and yields similar effects as longer treatment periods over several weeks. This advanced stimulation protocol may facilitate the access of rTMS in an outpatient setting as 5 days of treatment are easier to handle for PD patients with impaired mobility and may increase the efficacy of neuro‐rehabilitation in movement disorders.

## Conflict of Interest

MGE received research support from the German Ministry of Education and Research (BMBF) within the European Joint Program for Rare Diseases (EJP‐RD) 2021 Transnational Call for Rare Disease Research Projects (funding number 01GM2110), from the National Ataxia Foundation (NAF), and from Ataxia UK, and received consulting fees from Healthcare Manufaktur, Germany, all unrelated to this study. AF received travel support from CSL Behring, Ipsen, Ever Pharma and Indorsia, all unrelated to this project. AF and OK received research support from the German Parkinson's Association (Deutsche Parkinson Vereinigung), unrelated to this project. VB and TS declare no potential conflict of interest with respect to the research, the authorship and publication of this article.

## Author Contributions

OK conceived and designed the study. VB, MGE, AF, and TS contributed to the acquisition and analysis of the data. MGE and OK drafted a substantial portion of the manuscript or figures. All authors participated in editing of the manuscript.

## Supporting information


Figure S1.

Table S1.

## Data Availability

The data that support the findings of this study are available from the corresponding author upon reasonable request.

## References

[1] Armstrong MJ , Okun MS . Diagnosis and treatment of Parkinson disease: a review. JAMA. 2020;323:548‐560. 32044947 10.1001/jama.2019.22360

[2] Gong C , Long Y , Peng X‐M , et al. Efficacy and safety of noninvasive brain stimulation for patients with cerebellar ataxia: a systematic review and meta‐analysis of randomized controlled trials. J Neurol. 2023;270:4782‐4799. 37460852 10.1007/s00415-023-11799-8

[3] Sonmez AI , Camsari DD , Nandakumar AL , et al. Accelerated TMS for depression: a systematic review and meta‐analysis. Psychiatry Res. 2019;273:770‐781. 31207865 10.1016/j.psychres.2018.12.041 PMC6582998

[4] Shi R , Wang Z , Yang D , et al. Short‐term and long‐term efficacy of accelerated transcranial magnetic stimulation for depression: a systematic review and meta‐analysis. BMC Psychiatry. 2024;24:109. 38326789 10.1186/s12888-024-05545-1 PMC10851556

[5] Cole EJ , Stimpson KH , Bentzley BS , et al. Stanford accelerated intelligent neuromodulation therapy for treatment‐resistant depression. Am J Psychiatry. 2020;177:716‐726. 32252538 10.1176/appi.ajp.2019.19070720

[6] George MS , Wassermann EM , Kimbrell TA , et al. Mood improvement following daily left prefrontal repetitive transcranial magnetic stimulation in patients with depression: a placebo‐controlled crossover trial. Am J Psychiatry. 1997;154:1752‐1756. 9396958 10.1176/ajp.154.12.1752

[7] Yip AG , George MS , Tendler A , Roth Y , Zangen A , Carpenter LL . 61% of unmedicated treatment resistant depression patients who did not respond to acute TMS treatment responded after four weeks of twice weekly deep TMS in the Brainsway pivotal trial. Brain Stimul. 2017;10:847‐849. 28330592 10.1016/j.brs.2017.02.013

[8] Perera T , George MS , Grammer G , Janicak PG , Pascual‐Leone A , Wirecki TS . The clinical TMS Society consensus review and treatment recommendations for TMS therapy for major depressive disorder. Brain Stimul. 2016;9:336‐346. 27090022 10.1016/j.brs.2016.03.010 PMC5612370

[9] Lefaucheur J‐P , Aleman A , Baeken C , et al. Evidence‐based guidelines on the therapeutic use of repetitive transcranial magnetic stimulation (rTMS): an update (2014–2018). Clin Neurophysiol. 2020;131:474‐528. 31901449 10.1016/j.clinph.2019.11.002

[10] Li T , Le W , Jankovic J . Linking the cerebellum to Parkinson disease: an update. Nat Rev Neurol. 2023;19:645‐654. doi:10.1038/s41582-023-00874-3 37752351

[11] Wu T , Hallett M . The cerebellum in Parkinson's disease. Brain J Neurol. 2013;136:696‐709. 10.1093/brain/aws360 PMC7273201 23404337

[12] Basaia S , Agosta F , Francia A , et al. Cerebro‐cerebellar motor networks in clinical subtypes of Parkinson's disease. NPJ Parkinsons Disease. 2022;8:113. 10.1038/s41531-022-00377-w PMC9448730 36068246

[13] Appel‐Cresswell S , de La Fuente‐Fernandez R , Galley S , et al. Imaging of compensatory mechanisms in Parkinson's disease. Curr Opin Neurol. 2010;23:407‐412. 20610991 10.1097/WCO.0b013e32833b6019

[14] Kaut O , Mielacher C , Hurlemann R , Wüllner U . Resting‐state fMRI reveals increased functional connectivity in the cerebellum but decreased functional connectivity of the caudate nucleus in Parkinson's disease. Neurol Res. 2020;42:62‐67. 31900094 10.1080/01616412.2019.1709141

[15] Kawabata K , Watanabe H , Bagarinao E , et al. Cerebello‐basal ganglia connectivity fingerprints related to motor/cognitive performance in Parkinson's disease. Parkinsonism Relat Disord. 2020;80:21‐27. 32932024 10.1016/j.parkreldis.2020.09.005

[16] Sako W , Abe T , Furukawa T , et al. Differences in the intra‐cerebellar connections and graph theoretical measures between Parkinson's disease and multiple system atrophy. J Neurol Sci. 2019;400:129‐134. 30928779 10.1016/j.jns.2019.03.022

[17] Hacker CD , Perlmutter JS , Criswell SR , Ances BM , Snyder AZ . Resting state functional connectivity of the striatum in Parkinson's disease. Brain J Neurol. 2012;135:3699‐3711. 10.1093/brain/aws281 PMC3525055 23195207

[18] Scherbaum R , Hartelt E , Kinkel M , Gold R , Muhlack S , Tönges L . Parkinson's disease multimodal complex treatment improves motor symptoms, depression and quality of life. J Neurol. 2020;267:954‐965. 31797086 10.1007/s00415-019-09657-7

[19] Rascol O , Sabatini U , Fabre N , et al. The ipsilateral cerebellar hemisphere is overactive during hand movements in akinetic parkinsonian patients. Brain J Neurol. 1997;120(Pt 1):103‐110. 10.1093/brain/120.1.103 9055801

[20] Palmer SJ , Li J , Wang ZJ , McKeown MJ . Joint amplitude and connectivity compensatory mechanisms in Parkinson's disease. Neuroscience. 2010;166:1110‐1118. 20074617 10.1016/j.neuroscience.2010.01.012

[21] Yu H , Sternad D , Corcos DM , Vaillancourt DE . Role of hyperactive cerebellum and motor cortex in Parkinson's disease. NeuroImage. 2007;35:222‐233. 17223579 10.1016/j.neuroimage.2006.11.047 PMC1853309

[22] Koch G , Brusa L , Carrillo F , et al. Cerebellar magnetic stimulation decreases levodopa‐induced dyskinesias in Parkinson disease. Neurology. 2009;73:113‐119. 19597133 10.1212/WNL.0b013e3181ad5387

[23] Sanna A , Follesa P , Puligheddu M , et al. Cerebellar continuous theta burst stimulation reduces levodopa‐induced dyskinesias and decreases serum BDNF levels. Neurosci Lett. 2020;716:134653. 31778767 10.1016/j.neulet.2019.134653

[24] Bologna M , Di Biasio F , Conte A , et al. Effects of cerebellar continuous theta burst stimulation on resting tremor in Parkinson's disease. Parkinsonism Relat Disord. 2015;21:1061‐1066. 26117437 10.1016/j.parkreldis.2015.06.015

[25] Janssen AM , Munneke MAM , Nonnekes J , et al. Cerebellar theta burst stimulation does not improve freezing of gait in patients with Parkinson's disease. J Neurol. 2017;264:963‐972. 28382420 10.1007/s00415-017-8479-y PMC5413528

[26] Hughes AJ , Daniel SE , Kilford L , Lees AJ . Accuracy of clinical diagnosis of idiopathic Parkinson's disease: a clinico‐pathological study of 100 cases. J Neurol Neurosurg Psychiatry. 1992;55:181‐184. 1564476 10.1136/jnnp.55.3.181 PMC1014720

[27] Pickering RM , Grimbergen YAM , Rigney U , et al. A meta‐analysis of six prospective studies of falling in Parkinson's disease. Movement Disorders. 2007;22:1892‐1900. 17588236 10.1002/mds.21598

[28] Lamb SE , Jørstad‐Stein EC , Hauer K , Becker C , on behalf of the Prevention of Falls Network Europe and Outcomes Consensus Group . Development of a common outcome data set for fall injury prevention trials: the prevention of falls network Europe consensus. J Am Geriatr Soc. 2005;53:1618‐1622. 16137297 10.1111/j.1532-5415.2005.53455.x

[29] Allen NE , Schwarzel AK , Canning CG . Recurrent falls in Parkinson's disease: a systematic review. Parkinsons Disease. 2013;2013:906274. 10.1155/2013/906274 PMC3606768 23533953

[30] Oliveri M , Koch G , Torriero S , Caltagirone C . Increased facilitation of the primary motor cortex following 1 Hz repetitive transcranial magnetic stimulation of the contralateral cerebellum in normal humans. Neurosci Lett. 2005;376:188‐193. 15721219 10.1016/j.neulet.2004.11.053

[31] Hauser RA , Auinger P , Oakes D . Levodopa response in early Parkinson's disease. Movement Disorders. 2009;24:2328‐2336. 19908302 10.1002/mds.22759

[32] Chung CL‐H , Mak MK‐Y , Hallett M . Transcranial magnetic stimulation promotes gait training in Parkinson disease. Ann Neurol. 2020;88:933‐945. 32827221 10.1002/ana.25881 PMC8470277

[33] Manor B , Greenstein PE , Davila‐Perez P , Wakefield S , Zhou J , Pascual‐Leone A . Repetitive transcranial magnetic stimulation in spinocerebellar ataxia: a pilot randomized controlled trial. Front Neurol. 2019;10:73. 30809184 10.3389/fneur.2019.00073 PMC6380199

[34] Li R , He Y , Qin W , et al. Effects of repetitive transcranial magnetic stimulation on motor symptoms in Parkinson's disease: a meta‐analysis. Neurorehabil Neural Repair. 2022;36:395‐404. 35616427 10.1177/15459683221095034

[35] Balash Y , Peretz C , Leibovich G , Herman T , Hausdorff JM , Giladi N . Falls in outpatients with Parkinson's disease: frequency, impact and identifying factors. J Neurol. 2005;252:1310‐1315. 15895303 10.1007/s00415-005-0855-3

[36] Ilg W , Giese MA , Gizewski ER , Schoch B , Timmann D . The influence of focal cerebellar lesions on the control and adaptation of gait. Brain J Neurol. 2008;131:2913‐2927. 10.1093/brain/awn246 18835866

[37] Morton SM , Bastian AJ . Mechanisms of cerebellar gait ataxia. Cerebellum (London, England). 2007;6:79‐86. 17366269 10.1080/14734220601187741

[38] Timmann D , Brandauer B , Hermsdörfer J , et al. Lesion‐symptom mapping of the human cerebellum. Cerebellum (London, England). 2008;7:602‐606. 18949530 10.1007/s12311-008-0066-4

[39] Shimizu H , Tsuda T , Shiga Y , et al. Therapeutic efficacy of transcranial magnetic stimulation for hereditary spinocerebellar degeneration. Tohoku J Exp Med. 1999;189:203‐211. 10674722 10.1620/tjem.189.203

[40] Shiga Y , Tsuda T , Itoyama Y , et al. Transcranial magnetic stimulation alleviates truncal ataxia in spinocerebellar degeneration. J Neurol Neurosurg Psychiatry. 2002;72:124‐126. 11784843 10.1136/jnnp.72.1.124 PMC1737717

[41] Benussi A , Pascual‐Leone A , Borroni B . Non‐invasive cerebellar stimulation in neurodegenerative ataxia: a literature review. Int J Mol Sci. 2020;21:21. 10.3390/ijms21061948 PMC7139863 32178459

[42] Xia Y , Wang M , Zhu Y . The effect of cerebellar rTMS on modulating motor dysfunction in neurological disorders: a systematic review. Cerebellum (London, England). 2023;22:954‐972. 36018543 10.1007/s12311-022-01465-6

[43] Nardone R , Versace V , Brigo F , et al. Transcranial magnetic stimulation and gait disturbances in Parkinson's disease: a systematic review. Neurophysiologie clinique =. Clin Neurophysiol. 2020;50:213‐225. 10.1016/j.neucli.2020.05.002 32620273

[44] Peterchev AV , Luber B , Westin GG , Lisanby SH . Pulse width affects scalp sensation of transcranial magnetic stimulation. Brain Stimul. 2017;10:99‐105. 28029593 10.1016/j.brs.2016.09.007 PMC5241181

[45] Deng Z‐D , Lisanby SH , Peterchev AV . Coil design considerations for deep transcranial magnetic stimulation. Clin Neurophysiol. 2014;125:1202‐1212. 24411523 10.1016/j.clinph.2013.11.038 PMC4020988

[46] Chou Y , Hickey PT , Sundman M , Song AW , Chen NK . Effects of repetitive transcranial magnetic stimulation on motor symptoms in Parkinson disease: a systematic review and meta‐analysis. JAMA Neurol. 2015;72:432‐440. 25686212 10.1001/jamaneurol.2014.4380 PMC4425190

[47] Yang C , Guo Z , Peng H , et al. Repetitive transcranial magnetic stimulation therapy for motor recovery in Parkinson's disease: a meta‐analysis. Brain Behav. 2018;8:e01132. 30264518 10.1002/brb3.1132 PMC6236247

[48] Lynch G , Kramár EA , Babayan AH , Rumbaugh G , Gall CM . Differences between synaptic plasticity thresholds result in new timing rules for maximizing long‐term potentiation. Neuropharmacology. 2013;64:27‐36. 22820276 10.1016/j.neuropharm.2012.07.006 PMC3445784

[49] Nettekoven C , Volz LJ , Kutscha M , et al. Dose‐dependent effects of theta burst rTMS on cortical excitability and resting‐state connectivity of the human motor system. J Neurosci. 2014;34:6849‐6859. 24828639 10.1523/JNEUROSCI.4993-13.2014 PMC4019799

[50] Herculano‐Houzel S . The human brain in numbers: a linearly scaled‐up primate brain. Front Hum Neurosci. 2009;3:31. 19915731 10.3389/neuro.09.031.2009 PMC2776484

[51] de Zeeuw CI , Lisberger SG , Raymond JL . Diversity and dynamism in the cerebellum. Nat Neurosci. 2021;24:160‐167. 33288911 10.1038/s41593-020-00754-9

[52] Bostan AC , Strick PL . The basal ganglia and the cerebellum: nodes in an integrated network. Nat Rev Neurosci. 2018;19:338‐350. 29643480 10.1038/s41583-018-0002-7 PMC6503669

[53] Caligiore D , Arbib MA , Miall RC , Baldassarre G . The super‐learning hypothesis: integrating learning processes across cortex, cerebellum and basal ganglia. Neurosci Biobehav Rev. 2019;100:19‐34. 30790636 10.1016/j.neubiorev.2019.02.008

[54] Manto M , Argyropoulos GPD , Bocci T , et al. Consensus paper: novel directions and next steps of non‐invasive brain stimulation of the cerebellum in health and disease. Cerebellum (London, England). 2022;21:1092‐1122. 34813040 10.1007/s12311-021-01344-6

[55] Schmahmann JD , Sherman JC . The cerebellar cognitive affective syndrome. Brain J Neurol. 1998;121(Pt 4):561‐579. 10.1093/brain/121.4.561 9577385

[56] Manto M , Kakei S , Mitoma H . The critical need to develop tools assessing cerebellar reserve for the delivery and assessment of non‐invasive cerebellar stimulation. Cerebellum Ataxias. 2021;8:2. 33397496 10.1186/s40673-020-00126-w PMC7784008

[57] Formaggio E , Tonellato M , Antonini A , et al. Oscillatory EEG‐TMS reactivity in Parkinson disease. J Clin Neurophysiol. 2023;40:263‐268. 34280941 10.1097/WNP.0000000000000881

[58] Koch G , Esposito R , Motta C , et al. Improving visuo‐motor learning with cerebellar theta burst stimulation: behavioral and neurophysiological evidence. NeuroImage. 2020;208:116424. 31794855 10.1016/j.neuroimage.2019.116424

[59] Gassmann L , Gordon PC , Ziemann U . Assessing effective connectivity of the cerebellum with cerebral cortex using TMS‐EEG. Brain Stimul. 2022;15:1354‐1369. 36180039 10.1016/j.brs.2022.09.013

[60] Adamaszek M , D'Agata F , Ferrucci R , et al. Consensus paper: cerebellum and emotion. Cerebellum (London, England). 2017;16:552‐576. 27485952 10.1007/s12311-016-0815-8

[61] Jacobi H , Faber J , Timmann D , Klockgether T . Update cerebellum and cognition. J Neurol. 2021;268:3921‐3925. 33656586 10.1007/s00415-021-10486-w PMC8463403

