# Supplementary material for: Cerebellar transcranial magnetic stimulation improves motor function in Parkinson's disease
Source: Ann Clin Transl Neurol. 2024 Sep 5;11(10):2673–84. doi: 10.1002/acn3.52183 (PMC11514926; doi:10.1002/acn3.52183)
Supplement: Supplementary file 1 — Figure S1. [file ACN3-11-2673-s001.docx]

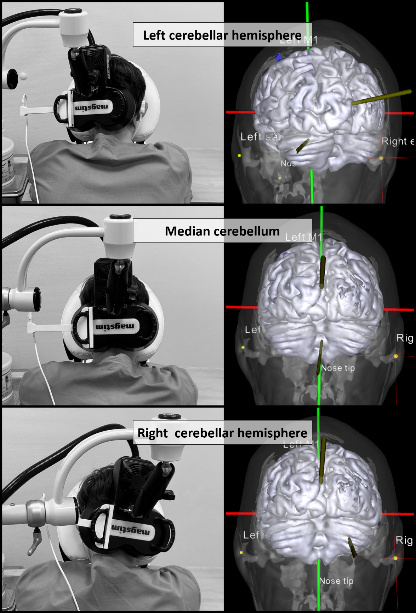


**Figure S1:** Photos of coil positions (left) and images of stimulation targets (right) for cerebellar transcranial magnetic stimulation. Targets were stimulated in the same order in each session: left cerebellar hemisphere, median cerebellum, right cerebellar hemisphere.


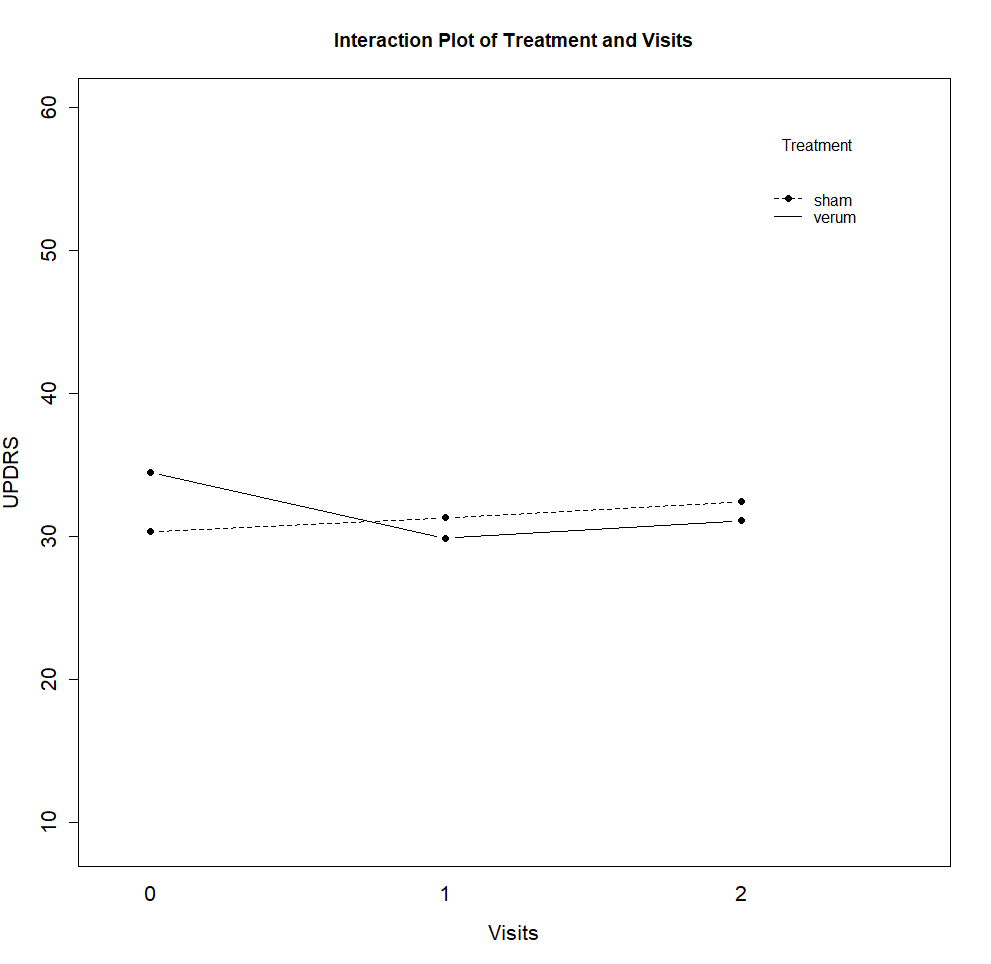


**Figure S2:** Interaction plot from two-way mixed ANOVA with repeated measures of UPDRS III. The plot provides a visual representation of the relationship between treatment, visits, and UPDRS scores. The descending trendline of the verum group between V0 and V1 represents amelioration of disease severity as measures by UPDRS III. In contrast, the trendline of the sham group shows no improvement.


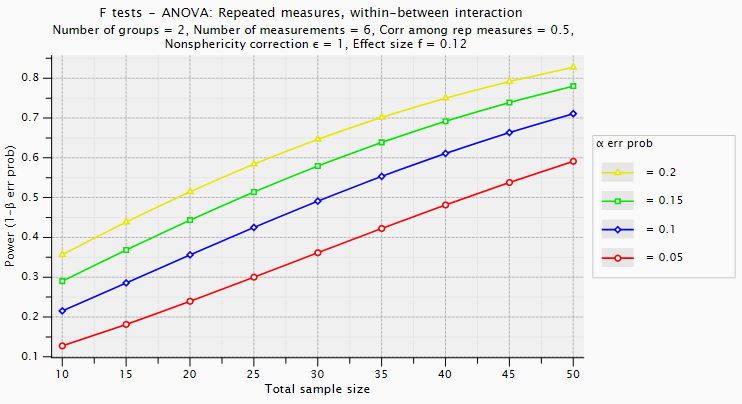


**Figure S3:** Post-hoc power analysis for the two-way mixed ANOVA with repeated measures of UPDRS III. Significance level of α = 0.1, effect size f = 0.12.


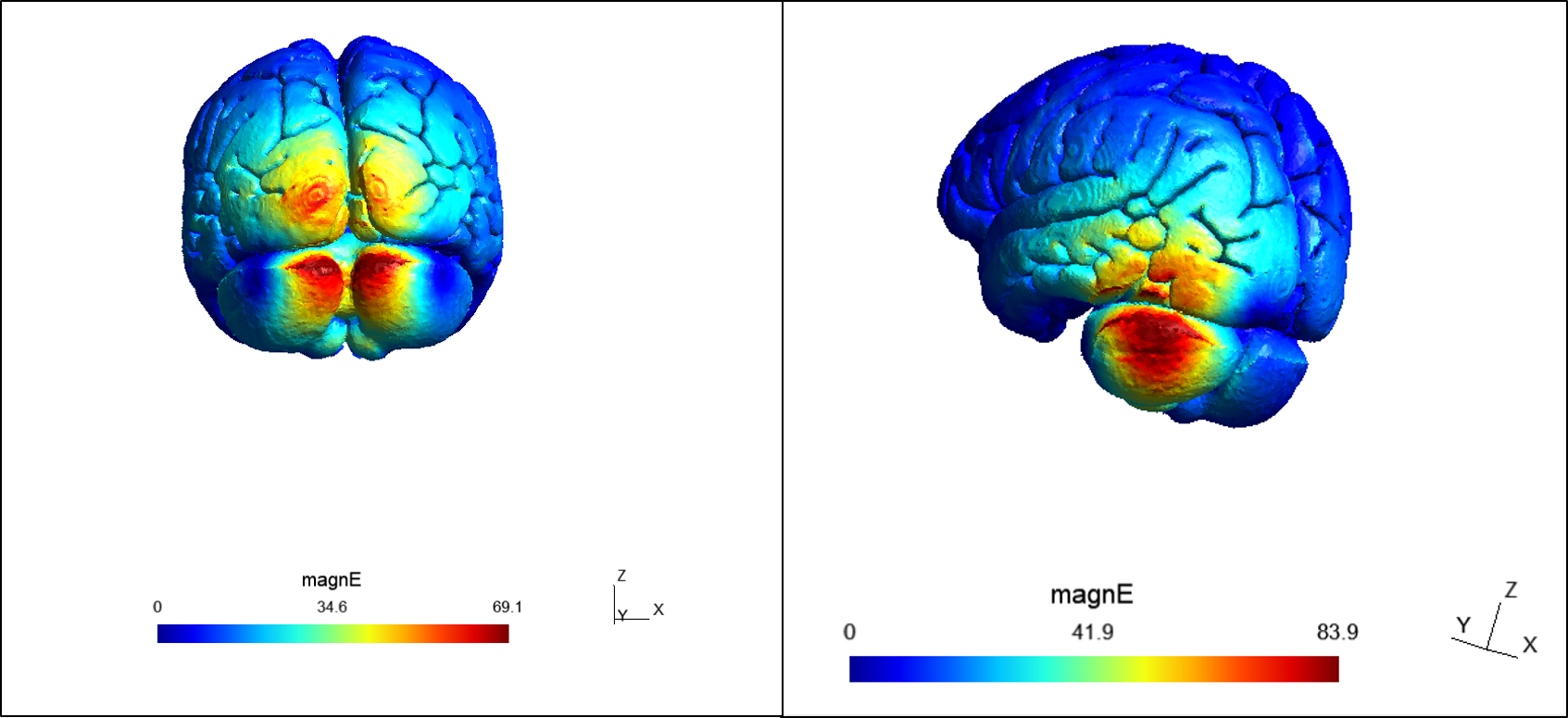


**Figure S4:** Electric field estimation (SimNIBs) according to the study protocol indicates that the cerebellar hemispheres and (to a lesser extent) the vermis are principally reached by the induced electric field.
