# Supplementary material for: Cerebellar transcranial magnetic stimulation improves motor function in Parkinson's disease
Source: Ann Clin Transl Neurol. 2024 Sep 5;11(10):2673–84. doi: 10.1002/acn3.52183 (PMC11514926; doi:10.1002/acn3.52183)
Supplement: Supplementary file 2 — Table S1. [file ACN3-11-2673-s002.docx]

| **Baseline** | **sham** | **verum** | **p** | **SMD** |
| --- | --- | --- | --- | --- |
| **n** | 17 | 18 |  |  |
| **age (mean (SD))** | 70.41 (10.37) | 66.06 (9.70) | 0.215 | 0.433 |
| **AOO (mean (SD))** | 63.35 (12.15) | 59.22 (10.42) | 0.287 | 0.365 |
| **UPDRS (mean (SD))** | 30.12 (14.65) | 34.17 (14.02) | 0.409 | 0.282 |
| **TUG (mean (SD))** | 8.98 (2.89) | 9.95 (5.28) | 0.506 | 0.229 |
| **8MW (mean (SD))** | 5.71 (1.60) | 5.75 (1.93) | 0.954 | 0.020 |
| **Posturo (mean (SD))** | 251.87 (72.52) | 254.78 (58.27) | 0.899 | 0.044 |

**Supplementary Table 1:** Comparison of baseline characteristics between the verum and sham groups (t-test). *Age of onset (AOO), Timed up and Go test (TUG), 8 meter walk test (8MW), Dynamic posturography (Posturo), Standardized mean difference (SMD, as measure of effect size, also known as Cohen's d).*
